# The Effect of Using a Rehabilitation Robot for Patients with Post-Coronavirus Disease (COVID-19) Fatigue Syndrome

**DOI:** 10.3390/s23198120

**Published:** 2023-09-27

**Authors:** Tomasz Trzmiel, Renata Marchewka, Anna Pieczyńska, Ewa Zasadzka, Igor Zubrycki, Dominika Kozak, Michał Mikulski, Anna Poświata, Sławomir Tobis, Katarzyna Hojan

**Affiliations:** 1 Department of Occupational Therapy, Poznan University of Medical Sciences, 60-781 Poznan, Polandstobis@ump.edu.pl (S.T.); khojan@ump.edu.pl (K.H.); 2 Neurorehabilitation Ward, Greater Poland Provincial Hospital, 60-480 Poznan, Poland; 3 Institute of Automatic Control, Lodz University of Technology, 90-537 Łódź, Poland; igor.zubrycki@p.lodz.pl; 4 Egzotech sp. z o.o., 44-100 Gliwice, Poland; dominika.kozak@egzotech.com (D.K.); michael.mikulski@egzotech.com (M.M.); anna.poswiata@egzotech.com (A.P.); 5 Department of Physiotherapy, University of Health Science, 85-067 Bydgoszcz, Poland; 6 Department of Rehabilitation, Greater Poland Cancer Centre, 61-866 Poznan, Poland

**Keywords:** SARS-CoV-2, exercises, hand grip strength, occupational therapy, physiotherapy

## Abstract

The aim of this study was to compare the effectiveness of traditional neurological rehabilitation and neurological rehabilitation combined with a rehabilitation robot for patients with post-COVID-19 fatigue syndrome. Eighty-six participants transferred from intensive care units due to post-viral fatigue after COVID-19 were randomly divided into two groups: the intervention group and the control group. The control group received standard neurological rehabilitation for 120 min a day, while the intervention group received the same neurological rehabilitation for 75 min a day, complemented by 45 min of exercises on the rehabilitation robot. The Berg scale, Tinetti scale, six-minute walking test, isokinetic muscle force test, hand grip strength, Barthel Index, and Functional Independence Measure were used to measure the outcomes. Both groups improved similarly during the rehabilitation. Between groups, a comparison of before/after changes revealed that the intervention group improved better in terms of Functional Independence Measure (*p* = 0.015) and mean extensor strength (*p* = 0.023). The use of EMG-driven robots in the rehabilitation of post-COVID-19 fatigue syndrome patients was shown to be effective.

## 1. Introduction

In recent years, a new global health problem emerged due to the novel coronavirus—SARS-CoV-2—causing coronavirus disease 2019 (COVID-19). This virus, which first appeared in China, has quickly spread worldwide, causing a global pandemic. In addition to the characteristic symptoms of respiratory tract infections, such as fever, cough, or fatigue, in the course of COVID-19, many other symptoms are likely to appear, including loss of taste and smell, sore throat, headache, muscle or joint pain, skin rash, nausea and vomiting, and dizziness [1]. In the vast majority of cases, the course of the disease is mild (80%), according to the WHO. However, some patients require hospitalization, and the condition might even lead to death. Importantly, not only the course of COVID-19 constituted a public health problem [2]. It is estimated that up to 20% of patients who had recovered suffered from long-term complications after contracting COVID-19 [3,4], which was also observed in patients with a mild course of the disease [5].

A similar phenomenon has also been reported for other viral diseases. Tansey et al. [6] showed that in the case of the SARS-CoV-1 virus epidemic, which took place in 2003, over half of the patients discharged from hospitals after treatment of the disease developed fatigue syndrome. Among the respondents, as many as 64% experienced fatigue after three months, and 60% even a year after discharge. Similar symptoms were also observed not only after other coronavirus infections but also for viruses such as Epstein–Barr virus, coxsackieviruses, and cytomegaloviruses [7]. Currently, there is no consensus on a term or definition in the scientific literature to describe a patient’s symptoms persisting after recovering from COVID-19. The most commonly used terms are “Long-COVID” and “Post-COVID Syndrome”; however, the definitions of these terms also vary. Raveendran et al. [8] use the term “Long-COVID” to describe both post-acute COVID-19 (the state between three weeks and three months from onset) and chronic COVID-19 (over three months from onset of the disease). The National Institute for Health and Care Excellence (NICE) guidelines proposed other definitions. “Acute COVID” is defined as a state where symptoms and signs are present for up to 4 weeks. “Post-COVID syndrome” is defined as a state where the symptoms last more than three months. According to these definitions, between “Acute-COVID” and “Post-COVID syndrome”, there is “Ongoing COVID”. The NICE guidelines define “Long-COVID” as a term that includes ongoing COVID and post-COVID syndrome [9]. Despite the lack of agreement on definitions in the literature, there is a consensus opinion that persistent symptoms after COVID-19 infection pose a challenge to modern medicine, particularly those related to fatigue and reduced ability to perform physical activity (or even activities of daily living).

Fatigue syndrome observed after COVID-19 is characterized by chronic fatigue, sleep problems, depressive symptoms, and myalgia [10]. The underlying pathomechanisms of such conditions remain unclear. Some theories point out that they may be caused by post-viral immune system alterations. However, there are still no sufficient data to support this statement [11]. Another hypothesis is that the infection alters the drainage of cerebrospinal fluid and lymph from the brain, which causes an accumulation of cerebrospinal fluid, leading to a form of intracranial hypertension [10]. This statement may be partially supported by the fact that patients with chronic fatigue syndrome who underwent cerebrospinal fluid drainage improved. Other authors suggest that post-COVID symptoms, including fatigue, may be related to the disfunction of both pulmonary cells and respiratory muscles as the SARS-CoV-2 virus uses the angiotensin-converting enzyme 2 to enter both kinds of cells. Persistent symptoms may be explained by changes in respiratory muscle function as patients improve after rehabilitation and manual therapy of the diaphragm [12]. Despite different hypotheses and ongoing research, there is no clear evidence on the identity of the cause of the observed fatigue syndrome; its etiology is probably multifactorial. There is a need for efficient ways to manage post-COVID fatigue and loss of independence. Currently, rehabilitation is considered one of the most effective therapeutic options [9].

Various rehabilitation approaches are used to manage post-COVID symptoms [13,14,15]. Some authors suggested pulmonary rehabilitation [14], while others suggested combined aerobic and resistance training [13]. In every case, rehabilitation should be individually tailored in regard to the patient’s abilities, state of health, fitness, and symptoms [9,15]. Patients’ ability to perform exercises may be altered differently in each person, so it is important to perform a proper assessment of a patient’s physical state and adjust exercises, their length, load, and frequency individually. It is also important to remember that aside from symptoms related to COVID-19, patients may also have other conditions, which further strengthens the need for an individual approach during the rehabilitation process.

Due to the growing workload of medical staff as well as the ever-increasing costs of rehabilitation, new methods of patient therapy are also sought, involving advanced medical devices that have the potential to reduce the burden on multidisciplinary rehabilitation teams. In light of the abovementioned problems, robotic devices could greatly improve the ability of healthcare to manage post-COVID patients and their symptoms [4,16]. Therefore, the aim of this study was to compare the effectiveness of traditional neurological rehabilitation against neurological rehabilitation combined with a rehabilitation robot for patients with post-COVID-19 fatigue syndrome.

## 2. Materials and Methods

A prospective randomized clinical trial was conducted in the Neuro-rehabilitation Ward of the Provincial Hospital in Poznan, Poland, between January and August 2022. The study has been registered with ClinicalTrials.gov, ID: NCT05130736.

Participants who were transferred directly from Intensive Care Units (ICUs) due to post-viral fatigue after experiencing a severe course of COVID-19 according to the criteria presented by Carod-Artal [17] were included. Among those, there were four main criteria, namely deterioration of the ability to perform tasks carried out before the disease, severe fatigue not improved by rest, discomfort after physical activity, and nonrestorative sleep, and two additional ones: cognitive impairment and orthostatic intolerance. At least three main criteria and one additional criterion had to be met for a patient to be included in the study. As patients were transferred straight from the ICU, we waived the condition that the above symptoms had to be present for more than six months. Patients were excluded if they presented any of the following: active medical condition (infections; tumors; rheumatological, metabolic, endocrine, autoimmunological, cardiovascular diseases), bipolar disorder, dementia, nutritional disorders, addiction to alcohol or psychoactive substances, severe obesity, overtraining. A total of 86 participants were recruited. Participants were randomly divided into two groups: the intervention group (IG) and the control group (CG).

The CG received standard neurological rehabilitation for 120 min a day, while the IG received the same neurological rehabilitation for 75 min a day, complemented by 45 min of exercises on the rehabilitation robot (Egzotech sp. z o.o., Gliwice, Poland). The rehabilitation robot was a device constructed to support physiotherapists and patients during the rehabilitation process. Due to the fact that the robot is EMG-driven, the device is able to help patients with relatively low muscle strength perform active exercises. Even if muscle contraction, due to its weakness, is not visible, electrodes applied above the muscles record changes in their activity during the contraction. Those signals trigger the actuator, which moves the movable parts (extensions) of the device. A limb of the patient is attached to those extensions by straps, which allows them to perform a movement (such as flexion and extension of the elbow) and control the movement even in a situation when normal movement will not be possible due to muscle weakness. The intervention was described in detail in a previous feasibility study [4].

### 2.1. Measurement

Several measurement tools were used to assess functional physical ability (Berg scale, Tinetti scale, six-minute walking test (6MWT)), muscle strength (isokinetic muscle force of elbow flexors and extensors, hand grip strength (GS)), and independence in activities of daily living (Barthel Index, Functional Independence Measure).

The Berg scale is used to assess balance and measures the static and dynamic balance using 14 motor tasks. Each task is scored from 0 to 4, where 0 means that the subject is not able to perform the task, and 4 is scored when the subject performed the task in the best way assigned to the task. The maximum total score is 56 points, and it indicates that the patient is fully independent in ambulation [18]. The Tinetti scale assesses balance in standing and sitting, and its results predict the risk of falls. It comprises 9 tasks. First, balance during sitting is assessed. Then, the patient must stand and maintain balance in three different positions. The ability to maintain balance is assessed in each position [19]. The 6MWT is used to measure physical exercise tolerance. It should be performed indoors, on a flat surface with a marked distance of 30 m. Patients are asked to walk the marked distance as many times as they are able for 6 min [20].

**Figure 1:**
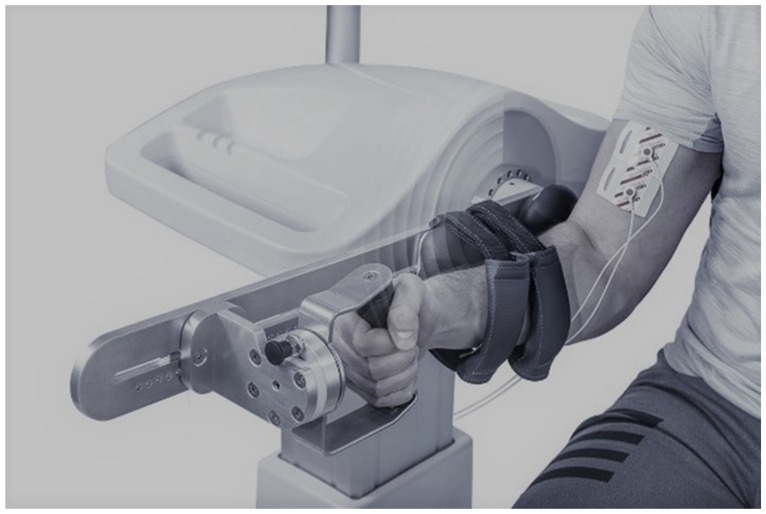
Participant upper limb position during elbow flexion/extension strength test (source—authors’ own).

The isokinetic muscle force test of elbow flexors and extensors in the dominant hand was performed on the rehabilitation robot. Patients were seated in a chair (dominant side toward the robot) with a straight back, hips and knees in 90 degrees of flexion, and feet on the floor. The dominant forearm was fixed by two straps and with the hand gripping a handle. The axis of the elbow was aligned with the axis of the dynamometer (Figure 1). Muscle force was assessed at 90 degrees of flexion in the elbow. Participants performed maximal voluntary concentric flexion and extension separated by a break of 1 min.

The HGS test was performed for the dominant hand using the JAMAR hydraulic hand dynamometer (Sammons Preston Rolyan, Bolingbrook, IL, USA). The test was performed in accordance with the gold standard of the American Society of Hand Therapists [21]. Participants sat straight on a chair without armrests, with knees and hips in 90-degree flexion and feet on the ground. The elbow was flexed to 90 degrees; the wrist was straight in intermediate rotation (thumb points to the ceiling). During the measurement, subjects were encouraged to squeeze the handle of the dynamometer as hard as possible. Contraction was maintained for 5 s, and three measures of dominant hand strength were conducted, with a 1 min break between each measure. The best result from the three measurements was taken for analysis.

The Barthel Index is a scale used to assess independence and the need for assistance in performing activities related to toileting, sphincter control, transferring to and from a wheelchair, and ambulation [22]. Functional independence was also assessed using the FIM. The FIM is an 18-item instrument that includes measures of independence in self-care, including ambulation, locomotion, communication, sphincter control, and cognition [23]. Each task of the FIM is scored from 1 point (which indicates that the subject is entirely dependent on assistance) to 7 points (which indicates that the subject is fully independent in performing the evaluated task).

### 2.2. Statistical Analysis

Statistical analysis was performed with Statistica 13.3 software (TIBCO Software/StatSoft Polska Sp. z o.o., Krakow, Poland). The threshold for statistical significance was set at *p* < 0.05. The Shapiro–Wilk test was used to check distribution normality. Student’s t-test was used to initially compare the results of the two groups. In the wake of the absence of a normal distribution, the Mann–Whitney U test was used. The ANOVA test for repeated measures was used to examine differences over time, taking into account the qualitative variable—group. The assumption of sphericity was checked with the Mauchley test; if sphericity was violated, the Greenhaus–Geisser correction was applied. Tukey’s test was used for post hoc analyses.

## 3. Results

**Figure 2:**
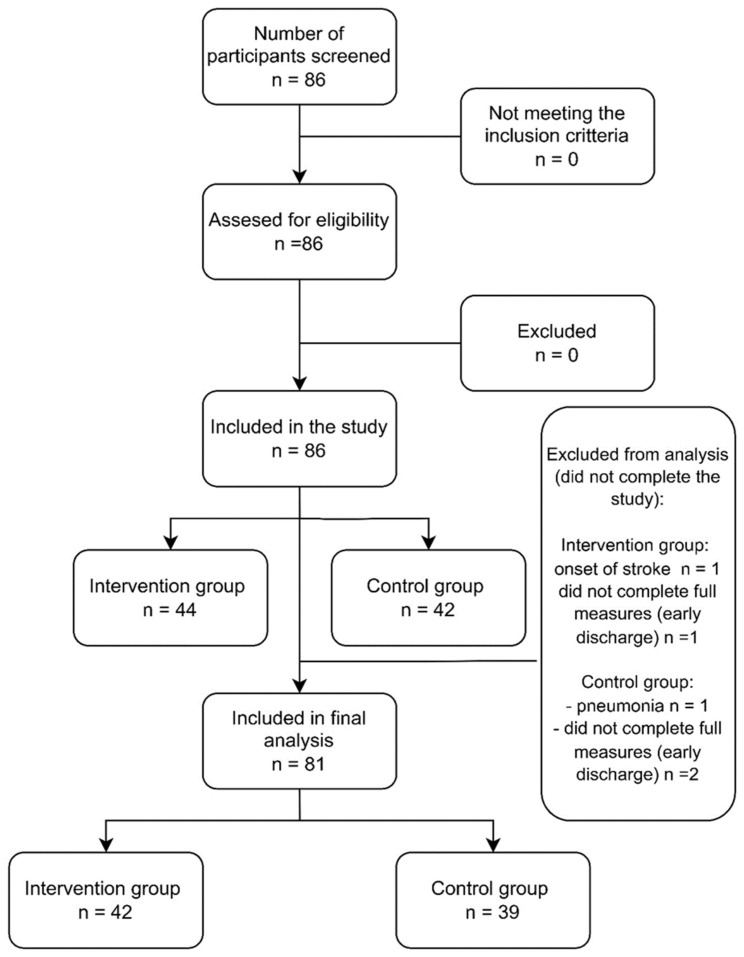
Study participant flowchart.

A total of 86 participants were included in the study. However, due to various causes, five participants did not complete the study. Therefore, data from 81 participants (42 in IG and 39 in CG) were analyzed. Figure 2 presents the flow diagram of the study.

There were significant differences between groups in terms of weight and BMI, yet groups did not differ in age, height, or any of the measured parameters at the baseline. Group characteristics comparison at the baseline is presented in Table 1.

**Figure 3:**
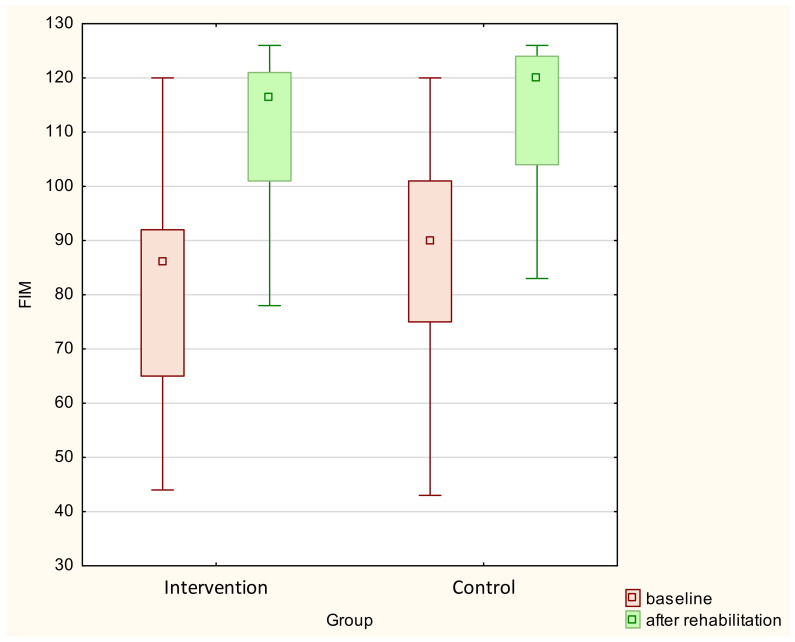
Comparison of baseline and post-rehabilitation FIM results.

**Figure 4:**
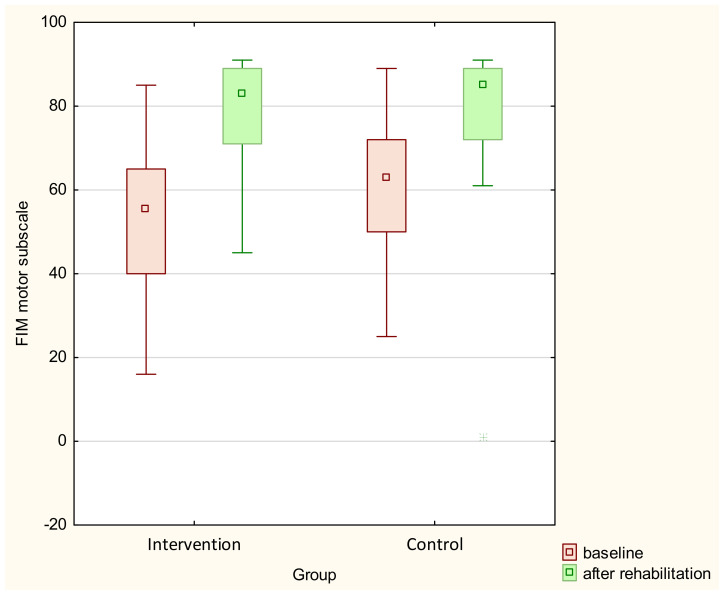
Comparison of baseline and post-rehabilitation FIM motor subscale results.

A comparison of the results before and after rehabilitation in both groups is presented in Table 2. In both the intervention and control groups, all parameters improved significantly over time. A comparison of pre- and post-rehabilitation FIM results is presented in Figure 3, and a comparison of its motor subscale is presented in Figure 4.

In the analysis of the results of repeated measures ANOVA, taking into account the division into groups, no intra-group interactions were found in any parameter except the FIM motor subscale (*p* = 0.047). The post hoc test showed differences between the FIM motor subscale before rehabilitation and the measurement after rehabilitation in both groups (<0.001).

## 4. Discussion

The use of robots in rehabilitation gained significant traction in 1990 [24], and this domain of medicine and technology continues to evolve. Robotic devices offer numerous advantages, including the ability to maintain precise and continuous movements, as well as the potential to engage users actively. These characteristics make them highly valuable in the rehabilitation process, forming the basis for the use of robots in various conditions, with stroke rehabilitation being particularly prominent. The vast majority of studies concerning rehabilitation robots focus on stroke patients; except for our previous pilot study, there are no available analyses comparing the effectiveness of robotic devices in the rehabilitation of patients with post-COVID-19 fatigue syndrome or other fatigue syndromes. The aim of this study was to compare the effectiveness of traditional neurological rehabilitation and neurological rehabilitation combined with a rehabilitation robot for patients with post-COVID-19-fatigue syndrome. We found that both groups improved similarly during the rehabilitation despite implementing different rehabilitation protocols. FIM is a tool that allows the measurement of independence in various domains. Some of the assessed tasks are not directly connected to upper limb function or physical function. In the present study, both groups improved after rehabilitation in all domains measured by FIM. ANOVA showed significant group × time interaction only in the summary score of the motor subscale. This result can be expected since the rehabilitation concentrated on the motoric aspect of patients’ functioning. However, the significance of this result is low (*p* = 0.047, almost on the verge of being insignificant), and post hoc analysis showed no statistical significance in the comparison of post-intervention measures between groups. Similarly, the Barthel scale, which measures a patient’s independence, showed significant improvements in both groups with no significant group × time interactions. These results show that both rehabilitation approaches lead to patients’ improvements in terms of independence, and neither approach is more or less beneficial than the other. The observed improvement in the FIM and Barthel scale should be considered clinically important, as changes in those scales, even by a few points, indicate meaningful changes in independence and can be related to decreased need for care [25]. These findings are coherent with the findings of other authors who compared traditional and robot-assisted rehabilitation [26,27,28].

Villafañe et al. [26] compared robot-assisted rehabilitation with traditional rehabilitation for patients with hand paralysis after stroke. Their study was conducted on thirty-two patients with hand paralysis after stroke who participated in rehabilitation. The control group received 90 min of traditional rehabilitation, while the study group received 60 min of traditional rehabilitation and 30 min of hand mobilization performed with a robotic device. Both groups improved, which led the authors to the conclusion that robotic interventions in rehabilitation should be supported. The present study shows comparable results with a similar methodology implemented. Supplementing part of traditional rehabilitation with robotic intervention did not negatively alter the improvement of patients with post-COVID fatigue syndrome. Mayr et al. [27] compared rehabilitation with gait training against rehabilitation with added robotic intervention to improve gait in patients after stroke. They found no significant differences between the study and control groups, while both groups improved from the baseline. Similar results were obtained in the present study. Rodgers et al. [28] compared the effects of robot-assisted training, enhanced upper limb therapy, and traditional rehabilitation on upper limb function after a stroke. Their study was conducted on 770 participants, and the results showed that robot-assisted training and enhanced upper limb therapy (as a substitute for traditional therapy) resulted in similar improvements. The abovementioned authors also obtained results similar to the results shown by the present study. However, Rodgers et al. did not recommend using robot-assisted rehabilitation due to the high cost of such interventions compared to usual therapy. The authors calculated that robot-assisted rehabilitation was the most costly form of treatment in their setting (GBP 5387 per participant), while traditional therapy cost was the lowest (GBP 3785 per participant). However, Bustamante-Valles et al. [29] obtained the opposite results. While they found that robot-assisted therapy and traditional rehabilitation after stroke were equally effective (which is coherent with our findings), they calculated that robot-assisted intervention was cheaper than traditional rehabilitation (6.99 vs. 19.21 USD/2 h, respectively). Unlike Rogers et al., Bustamante Valles et al. concluded that implementing robots in rehabilitation would improve the cost-effectiveness of interventions. This discrepancy can result from differences in economies, healthcare systems, and remuneration of health workers between Mexico and the UK. Another explanation of differences between those two studies can result from different algorithms of cost-effectiveness calculations. Bustamante Valles et al. [29] provided a detailed formula that was based on total expenses (yearly wage of one therapist, cost of purchase, and maintenance of robotic device) divided by the number of patients whom a single therapist was able to treat during one year. Rodgers et al. did not provide such details of their calculations. Another factor that could have impacted the cost-effectiveness of both interventions was a slight difference in setting. Rodgers et al. provided a robotic intervention in which one therapy assistant delivered a training program. The assistant was also supervised by a senior therapist, which required more staff than the Bustamante Valles et al. study. In the Bustamante Valles et al. study, one therapist was able to deliver therapy to a number of patients at the same time because patients were exercising on six stations of computer- and motor-assisted devices. Stations were distributed in a circle, which allowed the therapist to move between patients easily. As Carpino et al. [24] pointed out in a meta-analysis regarding lower limb rehabilitation after stroke, with and without robotic interventions, to measure the realistic cost of rehabilitation, trials should be designed differently than in the available literature. One should set a target for the patients to achieve instead of applying the same duration of rehabilitation with or without robotic devices. Such a scenario may show that with the use of robotic devices, rehabilitation goals will be achieved earlier in the traditional rehabilitation process, thus lowering the cost of patient rehabilitation (including side costs such as transportation, social care, hospital stay, and nursing care costs). Carpino et al. showed that rehabilitation using robots is more effective than classic rehabilitation, which is not confirmed by our present research. This may be due to the fact that these authors, in their meta-analysis, focused on robotic interventions for the lower limbs, while in our study, the robotic intervention concerned the upper limbs.

Some other authors found that robot-assisted rehabilitation can be more efficient than traditional rehabilitation. Dehem et al. [30] conducted a study on 28 stroke patients in which traditional upper limb rehabilitation was compared with combined rehabilitation and robotic-assisted therapy. The group that received combined therapy exhibited better improvement in terms of gross manual dexterity and upper limb ability during various functional tasks, while abilities to perform manual activities and activities of daily living presented similar improvements in both groups. The authors stated that rehabilitation combined with robot-assisted exercises was more effective than rehabilitation alone. Similar results were obtained by Daunoraviciene et al. [31] in a study conducted on 34 stroke patients, which aimed to identify the effect of robot training on the functional recovery of the arm. The authors used a device that was in the form of an orthosis with a system of springs to support the patient’s upper limb. The device also had sensors that continuously assessed the quality and range of the patient’s movement during training sessions and adjusted the degree of limb relief depending on the correctness of the performance of the motor task. The device did not use any actuators. The authors found that the study group (which underwent rehabilitation with additional robot-assisted exercises) presented significant improvement in upper extremity motor function compared to the control group (which underwent rehabilitation and occupational therapy sessions instead of robotic intervention). The abovementioned authors showed that robotic interventions in their studies were superior to traditional rehabilitation, which is inconsistent with our results. However, Dehem et al. showed that despite better improvement in the robotic group in terms of manual dexterity, the ability to perform manual tasks and activities of daily living improved similarly in both groups. This is partially in line with our results. The difference between the cited studies and the present one may result from the fact that in the present study, we did not use a detailed measurement of hand dexterity in terms of various functions.

As stated previously, the majority of available studies using robots in rehabilitation were conducted on stroke patients; however, a few authors compared using rehabilitation robots with conventional rehabilitation on patients with different conditions. Kawasaki et al. [32] and Pool et al. [33] assessed robotic assistive gait training for children with cerebral palsy. Kawasaki et al. included 11 children in their study; however, one of the participants resigned from the study because of the inability to accommodate the laboratory setting. The assistive device used in their trial comprised two tight frames that were attached to the patient’s legs and two actuators placed lateral to hip joints, which helped produce flexion and extension torque during the gait. The authors reported that in the studied group (n = 10), two parameters of gait–limb symmetry and the positive peak of the anterior–posterior ground reaction force improved, yet no change in gait speed was observed. Pool et al. used a different device in their study. Their robotic-assisted gait training used electrical stimulation of the participant’s muscles, helping them to actively increase the potential to withstand weight load by helping with a contraction of the participant’s own muscles. The authors demonstrated that adding robot-assisted gait training to locomotor training did not improve the gait performance of cerebral palsy patients. Both examples showed that different approaches are used to improve patients’ ability to move. Robotic devices are used not only to deliver external force to help start and maintain movement but also can improve muscle function by electrical stimulation. It is worth pointing out that Kawasaki et al. included children with Gross Motor Function Classification System (GMFCS) scores at levels I to III while Pool et al. included children with GMFCS levels III to V. This may further lead to different outcomes as the studied groups were at different physical performance levels at the baseline. Different results were obtained by Yildirim et al. [34] for patients with spinal cord injury. The authors used an exoskeleton with four motors and a computer. During the exercises, the device is capable of reducing the patient’s weight load, allowing the patient to walk as the weight of the patient is reduced. At the end of the training, patients were walking with full body weight (no assistance). The authors found that adding such robot-assisted gait training to rehabilitation improved the outcomes of gait reeducation in the studied group.

According to the available literature, rehabilitation using robots shows either similar effectiveness to classic rehabilitation, which is consistent with our results, or higher effectiveness than classic rehabilitation. The cost-effectiveness of this type of intervention remains an open issue that requires further research.

To our knowledge, the current study is the first clinical trial to compare the effectiveness of traditional neurological rehabilitation and neurological rehabilitation combined with a rehabilitation robot for patients with post-COVID-19 fatigue syndrome. We consider this as a strength of this study. However, the study has some limitations. Due to the setting, we are not able to calculate the realistic cost-effectiveness of the intervention because participants had to undergo the whole rehabilitation process, and rather than achieving the predetermined goal, their improvement was measured. A further limitation results from organizational and financial reasons, due to which only exercises of the upper limb were performed using the EMG-driven robotic device.

Human–robot interaction (HRI) is studied mainly in the area of social robotics. HRI may be different when a non-humanoid rehabilitation robot is used, like the one in the current study. Hertz and Wieze [35] showed that people tend to choose a human to interact with instead of a robot or screen applications. They also found that persons’ acceptance of a robot’s advice and compliance with its guidelines increased when robots looked more human-like. Tobis et al. demonstrated that a real-world interaction with a robot improved factors of its acceptance [36]. On the other hand, it has been established that people who suffer from physical illness are often subject to psychological distress [37], and actions conducted by physiotherapists to support and empower the patients are pointed out as important and beneficial in terms of physical and mental health [33,34,37,38]. In the present study, we measured neither the HRI nor the patients’ perceptions of robotic therapy. Psychological factors such as mood and self-acceptance were not assessed. Therefore, we consider this a third limitation. Further investigations should take into consideration how replacing an individual training with a physiotherapist with exercises on robotic devices impacts both the physical and mental domains of the patient. Another limitation is the unequal sample size. Differences in the size of groups result from the fact that we used a simple randomization. The study initially included a few more participants in both groups than required because we expected dropouts. However, differences in group sizes in the present study were small, and such differences are common in clinical trials even when groups are initially equal in size. The last limitation is that upper limb weakness was not an inclusion criterion. As most of the patients transferred from the ICU have some level of muscle weakness, we did not specify this criterion [39]. Different levels of weakness may potentially bias the results of the study; however, there were no statistical differences between the studied groups in terms of upper limb strength.

## 5. Conclusions

In light of the results of the present study, implementing EMG-driven robots in the rehabilitation of post-COVID-19 fatigue syndrome patients is effective. Substituting a part of traditional rehabilitation with robotic rehabilitation did not negatively alter rehabilitation outcomes. Our results indicate that the implementation of robotic rehabilitation for patients with post-coronavirus fatigue syndrome in rehabilitation programs can be supported. Further investigations aiming at achieving rehabilitation goals rather than completing a rehabilitation period should be conducted in the future to establish the realistic cost-effectiveness of such procedures.

## Figures and Tables

**Table 1:** Participants’ characteristics at baseline.

	Intervention Group n = 42		Control Group n = 39		*p*-Value
	Mean	SD	Mean	SD	
Age	67.38	8.46	64.92	11.74	0.280
Height	169.86	9.00	170.95	9.31	0.593
Weight	81.55	12.89	75.21	13.97	0.036
BMI	28.19	3.52	25.64	3.89	0.003
FIM	77.71	21.63	83.59	24.14	0.138
Berg	27.67	15.16	30.28	16.08	0.303
HGS	13.20	10.40	15.69	11.29	0.359
Tinetti	12.81	8.32	15.03	7.78	0.223
6MW	115.81	112.09	134.41	121.36	0.491
Barthel	10.48	3.97	11.00	3.10	0.762
Mean flexion strength	5.04	4.77	5.91	5.30	0.105
Peak flexion strength	16.22	10.35	18.40	12.34	0.397
Mean extension strength	4.75	3.83	5.95	4.51	0.157
Peak extension strength	15.31	9.11	16.65	10.52	0.674

BMI—body mass index; FIM—Functional Independence Measure; Berg—Berg scale; HGS—hand grip strength; Tinetti—Tinetti test; 6MWT—six-minute walking test; Barthel—Barthel Index; flexion strength—elbow flexion torque; extension strength—elbow extension torque.

**Table 2:** Repeated measures ANOVA results for all outcome measures.

	Intervention Group n = 42		Control Group n = 39		ANOVA *p*-Value		
	Pre	Post	Pre	Post	Time	Group	Group * Time
FIM	79.76 ± 18.32	110.60 ± 13.64	84.13 ± 24.15	108.62 ± 25.09	<0.001	0.78	0.076
FIM self-care	25.95 ± 8.68	37.24 ± 5.53	28.31 ± 10.55	36.31 ± 9.00	<0.001	0.679	0.055
FIM sphincter control	9.48 ± 2.94	12.71 ± 1.98	10.03 ± 3.33	12.38 ± 3.11	<0.001	0.851	0.097
FIM transverse	11.43 ± 4.59	18.33 ± 3.27	12.44 ± 5.83	18.05 ± 4.57	<0.001	0.434	0.325
FIM locomotion	5.45 ± 3.08	10.74 ± 2.72	6.26 ± 3.79	10.95 ± 3.15	<0.001	0.699	0.142
FIM motor subscale	52.31 ± 17.07	79.02 ± 12.30	57.03 ± 20.83	77.69 ± 19.22	<0.001	0.64	0.047
FIM communication	11.57 ± 2.34	13.10 ± 1.28	11.33 ± 2.31	12.87 ± 2.26	<0.001	0.57	0.975
FIM social cognition	15.88 ± 3.15	18.48 ± 2.23	15.77 ± 3.84	18.05 ± 4.39	<0.001	0.69	0.649
FIM cognition subscale	27.45 ± 5.07	31.57 ± 3.34	27.10 ± 5.83	30.92 ± 6.59	<0.001	0.638	0.778
Berg	27.67 ± 15.16	44.57 ± 12.83	30.28 ± 16.08	45.18 ± 14.25	<0.001	0.592	0.412
HGS	13.20 ± 10.40	18.08 ± 11.12	15.69 ± 11.29	20.22 ± 12.06	<0.001	0.342	0.768
Tinetti	12.81 ± 8.32	22.38 ± 5.89	15.03 ± 7.78	23.00 ± 5.91	<0.001	0.324	0.225
6MW	115.81 ± 112.09	240.02 ± 123.58	134.41 ± 121.36	256.69 ± 123.94	<0.001	0.483	0.919
Barthel	10.48 ± 3.97	17.95 ± 2.38	11.00 ± 3.10	17.44 ± 4.23	<0.001	0.996	0.123
Mean flexion strength	5.04 ± 4.77	7.42 ± 5.64	5.91 ± 5.30	6.97 ± 4.94	0.003	0.838	0.233
Peak flexion strength	16.22 ± 10.35	21.83 ± 11.52	18.40 ± 12.34	20.90 ± 11.16	<0.001	0.79	0.116
Mean extension strength	4.75 ± 3.83	7.36 ± 4.73	5.95 ± 4.51	16.65 ± 10.52	<0.001	0.633	0.118
Peak extension strength	15.31 ± 9.11	30.39 ± 26.08	6.99 ± 4.91	35.18 ± 29.92	<0.001	0.391	0.572

* Data are expressed as mean and standard deviation. BMI—body mass index; FIM—Functional Independence Measure; Berg—Berg scale; HGS—hand grip strength; Tinetti—Tinetti test; 6MWT—six-minute walking test; Barthel—Barthel Index; flexion strength—elbow flexion torque; extension strength—elbow extension torque.

## Data Availability

The authors confirm that the data supporting the findings of this study are available from the authors upon reasonable request.
